# Update on management of seminoma

**DOI:** 10.4103/0970-1591.60451

**Published:** 2010

**Authors:** Emma J. Alexander, Ingrid M. White, Alan Horwich

**Affiliations:** Royal Marsden Hospital, Downs Road, Sutton, Surrey, SM2 5PT, United Kingdom

**Keywords:** Carboplatin, chemotherapy, orchidectomy, radiotherapy, seminoma, surgery, surveillance, testicular germ cell tumors

## Abstract

Testicular germ cell tumors and, in particular, seminomas are exquisitely radiation and chemotherapy-sensitive and most presentations are highly curable. In recent years the management focus has been on reducing late sequelae of treatment. For Stage I disease surveillance and adjuvant carboplatin, chemotherapy has become an option. The efficacy of combination chemotherapy has been established for advanced metastatic disease. Through a review of the available literature this article outlines the recent changes in the management of seminoma.

## INTRODUCTION

Seminoma affects young men typically between the ages of 30 and 55 years and accounts for approximately 50% of testicular germ cell tumors (GCTs). It has high cure rates due to its extreme sensitivity to chemotherapy and radiotherapy. The metastatic pattern is relatively indolent and orderly in nature, spreading to the abdominal nodes, thereafter to pelvic and mediastinal nodes; visceral disease is a late and uncommon occurrence. Pure seminoma may be associated with a rise in blood levels of human chorionic gonadotrophin (HCG), which may also be raised in non-seminomatous germ cell tumors (NSGCTs); however, raised alphafetoprotein (AFP) would suggest a non-seminomatous origin and pathology should be reviewed carefully. As seminoma is a highly curable disease affecting a young population there are some survivorship issues to be considered in considering management; these include second malignancies, cardiovascular morbidity and fertility.

## STAGE I SEMINOMA

Approximately 80% of seminoma patients present with Stage I disease [Table 1].[1,2] It is the most common presentation of testicular cancer. Stage I seminoma had been managed with orchidectomy and adjuvant radiotherapy as the mainstay of treatment for over 50 years. As cure rates are high, in the order of 98–100%, the focus is now on reducing toxicity. This is particularly important for adjuvant treatment following orchidectomy, because about 80% of patients would not have needed any.[3] Accepted options for the management of recurrence risk in these patients also include surveillance and adjuvant carboplatin.

**Table 1:** Royal marsden hospital staging classification

RMH Stage	Classification	Description
I	M	Disease confined to the testis Rising markers post orchidectomy only
II	A	Transverse diameter of abdominal nodes <2 cm
II	B	Transverse diameter of abdominal nodes 2-5 cm
II	C	Transverse diameter of abdominal nodes >5 cm
III	A-C 0	Supra-diaphragmatic nodes (diameters as above) No abdominal nodes
IV		Extranodal Metastasis
	L1	≤3 lung metastases
	L2	>3 lung metastases, all <2cm
	L3	>3 lung metastases, one or more >2cm
	H +	Liver involvement

### Role of surgery

Radical inguinal orchidectomy is the operation of choice, to gain histology and for definitive local control. This involves ligation of the spermatic cord high in the internal inguinal ring. The procedure is generally well tolerated and associated with minimal post-operative complications. Retroperitoneal lymph node dissection (RPLND) is not performed as adjuvant treatment for seminoma.

### Role of radiotherapy

Traditionally, radiation portals encompassed the para-aortic, ipsilateral iliac and obturator nodes in a ‘dog-leg’ (DL) field to a dose of 30Gy. Although local control rates were excellent, long term data provided concern regarding late sequelae.

#### Toxicity from radiotherapy

Acute toxicity includes fatigue, nausea and mild emesis. Late toxicity includes peptic ulceration in 5%, oligospermia and increased risk of second cancers. Over the last decade there have been increasing questions regarding cardiac toxicity.[4–6]

Numerous data support an increased risk of second malignancies in testicular patients. The overall relative risk of second malignancy seems to be in the order of 1.2–2.0 for patients treated with adjuvant radiotherapy, with peak risk occurring between 10 and 20 years after treatment.[7–8] Risk increases inversely with age. Leukemia appears to be the most common non GCT malignancy (RR 4–6), followed by bladder, gastric and pancreatic tumors.[7,9] Some studies limiting data to infra-diaphragmatic radiotherapy find the risk to be lower and not statistically significant,[10] however the numbers in these studies are small.

Direct mediastinal irradiation has been shown to cause late cardiac toxicity in seminoma and other malignancies.[11–18] Data suggesting late cardiac effects from infra-diaphragmatic radiotherapy are also there. A large study (n=992) prospectively assessed testicular cancer patients for cardiac disease.[5] The relative risk of cardiac morbidity following radiotherapy alone was 2.74 compared with surveillance. After a median 10-year follow-up, more radiotherapy patients had suffered a cardiac event (9.6%) than those treated with chemotherapy (6.7%) or orchidectomy alone (3.7%). A recent larger epidemiological study (n=2,707), however, found conflicting results.[6] It showed an association between infra-diaphragmatic irradiation and increased risk of second malignancies (2.6-fold increase) but not with cardiovascular disease after a median follow-up of 17 years. Further long term follow-up is required to clarify the cardiovascular morbidity of infra-diaphramatic irradiation alone. It might relate to technique, such as inclusion of heart in the upper end of the para-aortic field, or to the extent of renal or renal vessel irradiation.

#### Randomized controlled trials

Data regarding second malignancies and cardiac toxicity originate from historic radiation techniques. Based on the assumption that the risk of these toxicities increase with increasing field size and dose; two Medical Research Council (MRC) trials conducted in the 1990s investigated limiting these factors whilst trying to maintain the high disease free survival (DFS).[19–20] The first of these trials (TE10) compared relative relapse rates and toxicity for a para-aortic (PA) strip and a dog-leg (DL) field at the then standard dose of 30Gy in 15 fractions over three weeks.[19] The reduction in field size had no impact on three-year relapse free survival (RFS) (96%) or overall survival (99–100%). Overall acute toxicity was lower in the PA strip group and oligospermia recovered more quickly. Pelvic recurrence was more common in the PA arm, but the total number of relapses was the same in both arms.

The second MRC trial in conjunction with the EORTC (TE18/EORTC 30942) looked at the efficacy and morbidity of a reduction in dose to 20Gy compared with the control of 30Gy.[20] The standard field was a PA strip. Acute toxicity and quality of life data were significantly improved at one month with the lower dose; with no significant difference by three months. Five-year RFS were similar (96–97%) and the actual difference in relapse rates was <1%. Recent updates of these trials have been presented and confirm low relapse rates with longer follow-up [Table 2].

**Table 2:** Updated results of MRC/ EORTC randomized trials

MRC/EORTC trial	No. of patients	Median follow-up	% followed to death or min 5 years	5-year DFS	HR
TE10	478	11 years	80	96.1% PA 96.2% DL	1.15 (90% CI 0.54-2.44)
TE18 / EORTC 30942	625	7 years	84	95.1% 30Gy 97.0% 20Gy	0.59 (90%CI 0.35-0.99)
TE19 / EORTC 30982	1447	6.5 years	78	96% radiotherapy 94.7% carboplatin	1.25 (90% CI 0.83-1.89)

[Mead *et al* J Clin Oncol 26: 2008 (May 20 suppl; abstr 5020)]

These trials have resulted in the evolution of standard portals of a PA field using 20 Gy in 10 fractions over two weeks. Resulting relapse rates are in the order of three to four percent[3,20,21] with the most common sites of relapse being the pelvic nodes[19,20,22] and the borders of the radiation field.[3,23] An annual pelvic CT is recommended for the first three years of follow-up.[24]

### Role of surveillance

Debate regarding overtreatment and late events following adjuvant radiotherapy led some centers to investigate surveillance as an option for Stage I seminoma as early as the 1980s.[25–27] Modern surveillance programs show the overall relapse rate to be in the order of 12–15% in the first three- four years with a 10-year overall relapse rate of approximately 18%–20%.[28–32] Therefore, approximately 80–85% of patients are likely not to require adjuvant therapy. Providing that disease is detected before it becomes bulky and due to the excellent results of salvage treatments, there appears no survival detriment. The patients, however, must be well motivated and compliant with the intensive follow-up.

### Risk factors for relapse

Research determining prognostic factors has helped characterize and select appropriate patients for surveillance programs. Multivariate analysis using pooled data from 638 patients[25–27,33] has shown two independent risk factors to be associated with a higher risk of relapse [1]: the presence of rete testis invasion and the size of the primary tumor (>4cm). The risk of relapse varied according to number of prognostic factors present. Patients with neither prognostic indicator had a 5 year relapse rate of 12.2%, those with one adverse factor, 15.9% and those with both adverse factors 31.5% (P<0.0001).[1] The five-year cause specific survival (CSS) for all surveillance patients in this study was 99.3%.

It is now common practice to use these prognostic indicators to counsel patients regarding the appropriate choice of therapy for them following orchidectomy. It must be cautioned, however, that these data have yet to be prospectively validated. A risk adapted strategy has been adopted by the Spanish Germ Cell Group using these criteria and the results have shown a relapse rate of 6.6% for patients with no risk factors who were observed.[34]

### Surveillance protocols

Surveillance programs vary, particularly, in terms of their imaging protocols; for example the Toronto schedule performs up to 20 CT scans whereas the Royal Marsden (RMH) schedule performs only 7. The optimum surveillance strategy is not known and there is little evidence to base decisions regarding the frequency of imaging. There does not seem to be a large difference in relapse rates in data from either of these institutions.[25,27] Our institution published its evidenced-based surveillance programs for testicular cancer follow-up in 2008.[35] For Stage I seminoma patients we recommend surveillance for those with zero or one risk factors and adjuvant therapy for patients with both risk factors present (RMH Surveillance Protocol Table 3), however, the patient is free to choose management after discussion.

**Table 3:** Royal marsden surveillance protocol for stage I seminoma patients

Year of Surveillance	Clinic and Tumor Markers	CT abdomen	CXR
1	3 monthly	6 monthly	6 monthly
2	3 monthly	6 monthly	6 monthly
3	4 monthly	Annual	Annual
4	6 monthly	Annual	Annual
5	6 monthly	Annual	Annual
6–10	Annual		

There is increasing concern regarding the malignant potential of ionizing radiation from regular CT surveillance. Mathematical modeling calculates the estimated lifetime risk of cancer from a single CT of the abdomen for a 25-year-old male as 0.06%.[36] A seven scan CT surveillance protocol may therefore carry a possible risk (assuming a linear relationship) of between 1 in 200 and 1 in 300 of a second malignancy related to imaging alone. A randomized MRC trial is currently investigating the frequency and modality of imaging in Stage I seminoma. It compares abdominal imaging in four arms using a 2x2 trial structure; seven CTs, three CTs, seven MRIs, or three MRIs.

### Role of chemotherapy

#### Efficacy

Phase II studies initially showed relapse rates from adjuvant carboplatin to be between 0–8.6 per cent [Table 4].[33,37–40] This led to a joint MRC /EORTC randomized trial (TE19/ EORTC30982).[21] With a 5:3 randomization, 885 patients received radiotherapy whilst 560 patients received carboplatin. Carboplatin patients received a single cycle dosed to achieve an area under the curve (AUC) of 7mg.mls per min, using the formula Dose = AUC × (GFR+25) where GFR is the Glomerular Filtration Rate.[41] An update of this study was presented in 2008 [Table 2]. Median follow-up was 6.5 years and relapse rates were similar; 5% for carboplatin vs. 4% for radiotherapy. Five-year CSS was 99.9% for radiotherapy and 100% for carboplatin. Carboplatin was associated with less fatigue and less time off work than radiotherapy. Results from the Spanish Germ Cell Group[34] using a risk-adapted policy show a recurrence rate of 3.8% in the carboplatin treated patients after three years follow-up. Their strategy was to give patients with one or both risk factors, two cycles of adjuvant carboplatin at AUC 7.

**Table 4:** Results of adjuvant carboplatin studies in stage I seminoma

Authors	No. of cases	No. of cycles	Dose of chemotherapy	Average FU months	Relapses (%)
Krege *et al*,[38]	43	2	400mg/m^2^*q21	28	0%
Dieckmann *et al*,[40]	93	1	400mg/m^2^	48	1 cycle 8.6%
	32	2	400mg/m^2^ q28		2 cycles 0%
Oliver *et al*	146	1	AUC 7	52	1 cycle 0.7%
J Clin Oncol 2001; 20 (suppl): 196a (abstr 780)	57	2	400mg/m^2^ q21	128	2 cycles 3.5%
Reiter *et al*[39]	107	2	400mg/m^2^ q21–28	74	0%
Steiner *et al*,[37]	108	2	400mg/m^2^ q21	60	1.9%
Aparicio *et al*,[99]	60	2	400mg/m^2^ q28	52	3.3%
Aparicio *et al* [J Clin Oncol 2004; 23 (suppl): 385 (abstr 4518)]	204	2	AUC 7 q21	20	2.4%
Oliver *et al* J Clin Oncol 26: 2008 (May 20 suppl; abstr 1)	573	1	AUC 7	78	5%
Powles *et al*,[43]	28	2	400mg/m^2^ q21	108	Whole group
	171	1	AUC 7		2%

* q = dosing schedule in days

#### Toxicity and dose

Combination cisplatin-based chemotherapy has been shown be associated with second malignancies, cardiac toxicity and vascular effects such as Raynaud's phenomena.[4,5] Caution regarding long term toxicity is advised as numbers are small and long term data are scant, although this does not appear to be the case with single agent carboplatin. One study has shown carboplatin to be associated with a modest increase in second malignancies in ovarian cancer.[42] A single institution series has reported 20 years experience with adjuvant carboplatin in Stage I seminoma.[43] Nearly 200 patients were followed up with no evidence of late toxicity. Despite a median follow-up of nine years, however, these data are still immature. There remains debate with respect to the optimum dosing of adjuvant carboplatin, with some advocating two cycles.[34] There are, as yet, no data proving that two cycles have improved efficacy, although there are suggestions that dose intensity may be important.[21,43]

## METASTATIC SEMINOMA

### Low Volume Stage II Seminoma (Stage IIA and IIB)

Just under one-fifth of patients with seminoma present with Stage II disease [Table 1].[44] Optimal treatment for Stage II seminoma patients depends on size and bulk of disease. Bulky disease (>5 cm, Stage IIC) treated with radiotherapy is associated with renal toxicity and poorer efficacy, so is generally treated with multiagent cisplatin-based chemotherapy. It will therefore be discussed in the section on advanced disease (see below).

### Role of Radiotherapy

Stage II seminoma is rare and therefore data are mainly restricted to single institutional retrospective series [Table 5].[37–40] Evidenced-based practice is limited by small numbers of patients with data accrued over many years (often decades) and therefore subject to stage migration and changes in radiotherapy techniques. If restricted to recent results and RMH staging of IIA/ IIB only, relapse rates are in the order of 6–15%[45–47] and five-year CSS remains high (94–100%).[18,45,48]

**Table 5:** Results of radiotherapy series for stage II seminoma patients

Authors	Years of Recruitment	No. of Patients	RMH Stage	5 Year Relapse Rates (unless otherwise stated)	5 year cause- survival	5 yr OS
Lederman *et al*,[100]	1968-1984	37	IIA-C	IIA 7% (10 yr) IIB 0% (10 yr) IIC 25% (10 yr)	NA	77%
Bayens *et al*,[101]	1975-1985	44	IIA-C	23%	93%	91%
Hanks *et al*,[18]	1973-1974	107	IIA-C	4%	97%	95%
Lai *et al*,[47]	1964-1988	33	IIA	IIA 7%	97%	89%
Vallis *et al*,[46]	1974-1989	48	IIA-IIB	9.4%	96.1%	NA
Whipple *et al*,[102]	1966-1989	45	IIA-IIB	9%	98%	98%
Warde *et al*,[45]	1981-1993	80	IIA-IID*	11% (IIA/IIB) 44% (IIC-IID)	94%	94%
Classen *et al*,[48]	1991-1994	87	IIA-B	4.7% (IIA) 11.1% (IIB)	100%	99%
Weissbach *et al*,[103]	1986-1991	82	IIA-B	2.3% (IIA) 21.1% (IIB)	NA	NA

NA Data not avaliable,

* Modification of the RMH classification system (IIC 5-10cm, IID >10cm)[104]

### Extent of Fields

The target volume for Stage II seminoma includes the para-aortic nodes along with the ipsilateral pelvic nodes. The lower border of this field is traditionally the mid or lower obturator foramen. The German Testicular Cancer Study Group (GTCSG) published a succession of papers prospectively following up a cohort of 87 patients in a trial of reduced field dog-leg radiotherapy.[48–50] This is the only clinical trial performed in Stage II seminoma, to date, with respect to radiotherapy. The inferior border of the field was the superior acetabulum, macroscopic nodes were given a 2cm margin and there were no boosts given. Doses prescribed were 30Gy for Stage IIA and 36Gy for Stage IIB. Six-year relapse rates were 4.7% for IIA disease and 11.1% for Stage IIB. Six-year CSS was 100% for both groups. No peptic ulcer disease has been reported and so far no second malignancies noted. These data suggest that a reduction in the standard dog-leg field does not compromise outcome for these patients, none of whom relapsed in the pelvis.

### Dose

With a lack of randomized trials, a wide variety of doses are used for Stage II seminoma radiotherapy. Doses in the range of 26Gy to 40Gy have been used, the most common being those used in the recent German clinical trial noted above. Given the local control rates achieved in this trial and in some series using lower doses,[45,51] there may be scope for further dose reduction but this needs to be evaluated within the context of a clinical trial.

European consensus guidelines now recommend an ipsilateral dog-leg field with the inferior border reduced to the superior acetabulum.[52] A margin of 1–1.5 cm on diseased nodes is advised with dose prescriptions of 30Gy in IIA and 36Gy in IIB disease. Extension to the contralateral iliac, inguinal or scrotal region is not recommended (even with T3/T4 tumors, prior testicular mal-descent, scrotal or inguinal surgery) due to a lack of evidence base.

### Role of Chemotherapy

The role of chemotherapy for IIA/B disease is ill defined. Multiagent cisplatin-based chemotherapy is considered by some to be a reasonable alternative to irradiation, especially in the presence of relatively bulky disease i.e. Stage IIB. There is, however, significant acute toxicity and rare but serious additional late toxicities such as cardiovascular disease from cisplatin and the risk of leukemia from etoposide.

### Single Agent Carboplatin

Single agent carboplatin has shown poor results in Stage IIA and IIB seminoma (as also for more advanced seminoma – see below). A prospective phase II trial by the GTCSG was terminated early due to a high failure rate with an overall DFS of 82%.[53] Relapse rates did not differ between Stage IIA and IIB, therefore 3 cycles of carboplatin did not effectively eradicate even small volume (< 2cm) lymph node metastases in this study.

### Cisplatin-based Chemotherapy

A Spanish multi-center prospective observational study (n=78) of cisplatin-based chemotherapy in low volume Stage II seminoma has been reported.[54] These data revealed a high DFS with cisplatin-based chemotherapy using 4 cycles of E400P (400mg per cycle of etoposide plus cisplatin) or three cycles of BEP (bleomycin, etoposide and cisplatin). Five year DFS for the whole group was 90% (100% IIA, 87% IIB) and overall survival 95%. Toxicity was greater than for carboplatin regimes; Grade 3 or 4 hematological toxicities were reported in 10–15%, including 11% febrile neutropenia. Eight per cent had Grade 3 or 4 emesis. No significant late toxicity had been reported at six years. It should be noted that the dose intensity of the etoposide in this regime was lower than considered standard for nonseminoma.

### Combination of Carboplatin and Irradiation

Another alternative being investigated is the combination of Carboplatin and Radiation. Patients with Stage II A/B disease at our institution are treated with a single cycle of carboplatin followed by 30Gy in 15 fractions over three weeks to the involved PA strip only. Two cohorts have been reported, 33 patients treated from 1989–1996[55] and 26 patients treated from 1998–2006 (Gilbert *et al*. GU ASCO 2008 Abst 279). Disease-free survival rates were 96.9% (five- year) and 100% (three-year), overall survivals were 96.7% and 100% respectively. No additional toxicity is seen from the addition of carboplatin to radiotherapy. We believe the relative risks of second malignancies and cardiac toxicity may be favorable with this approach compared with combination cisplatin-based chemotherapy.[5,56,57]

## BULKY STAGE II AND ADVANCED SEMINOMA (STAGES IIC/ III/ IV)

### Role of Chemotherapy

Advanced seminoma is rare, accounting for 5–6% of seminoma patients. As a result there are only a small number of studies investigating optimal treatment. Evidence comes from this limited data and from the many studies of chemotherapy for NSGCTs, which is the more common indication for chemotherapy. As with earlier stages of disease, there is a fine balance between adequate treatment for cure, and over treatment with its associated toxicities. Treatment is therefore divided into two categories; good and intermediate prognostic groups. Advanced seminoma is extremely sensitive to treatment with multiagent cisplatin- based chemotherapy, with overall survival of 86% for good prognosis and 72% for intermediate prognosis disease [Table 6].[58]

**Table 6:** IGCCCG prognostic grouping classification for metastatic seminoma.[58]

Prognosis	5-Year Overall Survival	5-Year Progression Free Survival	Description
Good (90% metastatic seminoma)	86%	82%	Any primary site and No non-pulmonary visceral metastases and Normal AFP, any hCG, any LDH
Intermediate (10% metastatic seminoma)	72%	67%	Any primary site and Non-pulmonary visceral metastases and Normal AFP, any hCG, any LDH

### First Line Chemotherapy for Good Prognosis Disease

Standard treatment for good prognosis disease is with BEP. Randomized trials have shown three cycles to be equivalent to four cycles in these patients.[59–61] It has been noted that the same dose may be administered over three days instead of five with similar results in terms of efficacy and toxicity.[60,62] A number of studies have investigated substitution of cisplatin with carboplatin, because of its favorable toxicity profile. They have all demonstrated carboplatin to be inferior in terms of progression-free survival [63,64] and chemotherapy with carboplatin is therefore not recommended unless cisplatin treatment is contraindicated.

Bleomycin is associated with pulmonary toxicity which can be fatal. Older patients, smokers and those with renal impairment are particularly at risk and in these patients it is preferable to avoid bleomycin.[65] Data from NSGCTs suggests that 3 cycles of BEP is equivalent to four cycles of EP, provided the dose intensity of etoposide is sufficient (E500P).[66–67] Both are considered acceptable, however, treatment with three cycles of BEP is the preferred option. Other late toxicities associated with EP and BEP chemotherapy are nephrotoxicity, ototoxicity, neuropathy, leukemia and reduced fertility. As previously noted, late cardiovascular effects and second malignancies have also been noted with cisplatin-based chemotherapy.[5,7,57] Van den Belt-Dusebout's recent epidemiological study [57] revealed a 1.7-fold increased risk of cardiovascular disease associated with chemotherapy in testicular patients and a 2.1-fold increased risk in second malignancy.

### First Line Chemotherapy for Intermediate Prognosis Disease

More aggressive treatment is indicated for patients with non-pulmonary visceral metastases. In these patients, current evidence supports treatment with four cycles of BEP, which should be given over five rather than three days to avoid acute gastrointestinal toxicity and tinnitus.[62,68] An alternative is EP as discussed above, or for very advanced disease, especially if a nonseminomatous component is suspected, ifosfamide, in combination with etoposide and cisplatin (IPE), which is comparable with BEP in terms of response rates and survival, but is associated with increased myelosuppression and genitourinary toxicity.[69]

It is essential to limit dose reductions and delays to an absolute minimum to ensure optimal cure rates. Low blood counts do not necessarily require dose reductions unless they are associated with febrile neutropenia or other significant complications. Granulocyte colony-stimulating factors are not routinely used, but may maintain dose intensity if there have been serious infections or prolonged neutropenia in previous cycles.[70] The use of prophylactic antibiotic treatment during chemotherapy may reduce infection risk.

### Salvage Chemotherapy for Relapsed or Refractory Disease

Patients who relapse after first line treatment with radiotherapy have a good prognosis and should receive cisplatin-based chemotherapy as outlined above. Following first line chemotherapy for advanced germ cell tumors, approximately 15% will relapse.[44] Second line chemotherapy with further cisplatin-based regimens can result in long term disease free survival in up to 50% of patients.[71,72] The two combinations of choice are vinblastine, ifosfamide and cisplatin (VIP) or paclitaxel, ifosfamide and cisplatin (TIP); neither of these regimens has shown superiority with respect to each other.[73–74]

Given the small numbers, there is a paucity of data on treatment for relapse following salvage chemotherapy specifically for seminoma patients. Treatment is largely based on that for NSGCTs. High dose chemotherapy, supported by stem cell transplantation could be an option and carboplatin and etoposide are commonly used in this situation. In some studies a high cure rate has been achieved, albeit with significant mortality rates.[75–77] Other treatment options include gemcitabine, oxaliplatin and paclitaxel, which have been investigated both as single agents and in combination chemotherapy treatment.[78–80]

### Role of Surgery

Residual masses are common in seminoma, especially after treatment for initially bulky disease.[81] Up to 80% of patients may experience a residual mass on imaging over one month after completion of therapy.[82–84] Radical retroperitoneal lymph node dissection, whilst commonly employed in NSGCTs, is rarely used in seminoma patients due to increased desmoplastic reaction and fibrosis, thus significantly increasing the morbidity of the procedure.[85–89] Surgery in the form of biopsy or resection is therefore favored in this situation.

Most units treating GCTs have used a cut-off of 3 cm for considering biopsy for residual seminomatous masses. Observation, certainly in the early days after completion of chemotherapy, is a reasonable option. There are data that support the continued regression of seminoma masses, over months and sometimes over several years.[84,90] Viable tumor, however, may be seen in up to 30% of large residual seminomatous masses[84–85] and may be treated with salvage chemotherapy, which is effective in approximately 50% of cases[72] and/or radiotherapy.

Retrospective series show viable malignancy rates of 20–30% in residual masses over 3cm compared with 0% in masses under 3cm.[91–92] Appearances on CT scans affect the resectability. One study showed that 78% of discreet masses were resectable as opposed to 44% of poorly defined masses, which often include retroperitoneal fibrosis and scarring.[93] There have been four FDG-PET studies performed in seminomatous patients with post chemotherapy residual masses to date[94–97] [Table 7]. These have shown some promise for the utility of PET in terms of its negative predictive value, however, false positive results are a problem. It is still very much an area of research, however, and PET does not yet have an established role in seminoma.

**Table 7:** PET results for residual masses post chemotherapy in seminoma

Authors	No of residual masses	Specificity%	Sensitivity%	PPV%	NPV%
Ganjoo *et al*,[96]	29	96	0	0	83
De Santis *et al*,[95]	51	100	80	100	96
Becherer *et al*,[97]	74	100	80	100	95
Hinz *et al*,[94]	20	47	100	25	100

### Role of Radiotherapy following Chemotherapy

A retrospective study of 302 patients with residual masses has evaluated the role of radiation following chemotherapy treatment in advanced seminoma.[98] There was no improvement in progression-free survival with the routine addition of radiotherapy to platinum-based chemotherapy. Radiotherapy is therefore not routinely used in the first line treatment of advanced disease following chemotherapy. It ma, however, be considered in practice for residual masses following salvage chemotherapy for residual or relapsed disease, although there is little evidence base.

## SUMMARY

Seminoma may be treated using a variety of treatment modalities and is highly curable whichever modality is chosen. Data on long term morbidity has begun to shape management decisions for seminoma patients and there is continued research into minimizing toxicity in this group of patients. The focus for the future will be on improved risk stratification and tailoring of treatment for individual patients. Improvements in imaging e.g. nanoparticle MRI and PET may further improve staging and surveillance techniques and treatment modifications may be increasingly based on individual risk from pathological and molecular stratification.
